# TNFSF9 promotes metastasis of pancreatic cancer through Wnt/Snail signaling and M2 polarization of macrophages

**DOI:** 10.18632/aging.203497

**Published:** 2021-09-13

**Authors:** Jiao Wu, Yunpeng Wang, Yichun Yang, Fuqiang Liu, Jun Chen, Zhongxiang Jiang, Zheng Jiang

**Affiliations:** 1 Departments of Gastroenterology, The First Affiliated Hospital of Chongqing Medical University, Chongqing 400016, China; 2 Departments of Cardiovascular, Zigong First People’s Hospital, Sichuan 643000, China

**Keywords:** pancreatic cancer, TNFSF9, metastasis, macrophages, inflammatory factors

## Abstract

Early metastasis of pancreatic cancer (PC) leads to high mortality, and the underlying mechanism of metastasis remains unclear. Tumor necrosis factor superfamily member 9 (TNFSF9) is associated with poor prognosis in PC. Here, we investigated the effect of TNFSF9 on PC proliferation and apoptosis, and focused on the effect of TNFSF9 on PC metastasis and its potential mechanism. We found that TNFSF9 promotes PC metastasis *in vivo* and *in vitro*, and may be partially dependent on the Wnt/Snail signaling pathway. In addition, TNFSF9 also regulates the release of cytokines IL-10 and transforming growth factor-β (TGF-β) in pancreatic cancer cells through Wnt signaling to induce the M2 polarization of macrophages and promote the migration of PC cells. Overall, our study found that TNFSF9 may directly promote PC metastasis or indirectly promote PC metastasis through macrophage M2 polarization. Our study provides a new costimulatory target for the treatment of PC.

## INTRODUCTION

Pancreatic cancer is one of the most deadly and incurable cancer types, and its 5-year survival rate is less than 10% [1]. Among the most common malignant tumors diagnosed in men and women in 2021, their incidence is the 10th and 8th respectively. But their mortality rates are ranked 4th [2]. Despite all efforts, this malignancy is still difficult to diagnose in time and prone to recurrence and metastasis [3]. Therefore, it is very important to actively explore the mechanism of PC development for the formulation of pancreatic cancer treatment.

Previous researchers have found that PC has a poor response to immune checkpoint inhibitors, but patients with positive predictive biomarkers have better clinical response [4, 5]. We previously found that the expression of TNFSF9 is significantly increased in PC, and its expression is negatively correlated with the survival rate of PC [6]. Therefore, TNFSF9 may play a role as a predictive marker of PC. TNFSF9 was originally found to be expressed on antigen-presenting cells such as macrophages. Recent studies have shown that TNFSF9 is not only expressed on antigen-presenting cells, but also observed in solid tumors such as adenocarcinoma and squamous cell carcinoma, especially in moderate or poorly differentiated tumors [7–10]. The expression of TNFSF9 found on tumor cell lines is functional and has different functions in various types of cells [11]. In colon cancer patients, TNFSF9 expression is highly upregulated in early to late stage tumors and is significantly associated with the occurrence of distant metastases and shortened survival in late disease [8]. In breast cancer, TNFSF9 is cross-linked with TNFRSF9, which promotes monocyte/macrophage migration to the tumor microenvironment and osteoclast generation by upregulation of FRA1 expression, thereby promoting breast cancer metastasis [12]. However, the role of TNFSF9 in the development of PC has not been studied.

In addition, TNFSF9 was widely expressed in lung cancer cell lines at mRNA level, while the protein level was generally low. However, low expression of TNFSF9 protein was still sufficient to induce T cells to produce Interferon-γ (IFN-γ), followed by increased expression of programmed death ligand 1 in lung cancer cells [9]. Furthermore, the connection of TNFSF9 on lung squamous cell carcinoma cells L78 and TNFRSF9 on T cells can induce T cells to produce IFN-γ [7]. The cross-linking of TNFSF9 and TNFRSF9 on liver cancer cells HepG2 triggers the production of interleuukin-8 (IL-8) by tumor cells [7]. It can be seen that the increased expression of TNFSF9 on tumor cells also regulates the release of cytokines in the tumor microenvironment. Therefore, we speculate that in PC, TNFSF9 may affect the occurrence and development of PC by regulating the release of cytokines in the microenvironment.

Increasing evidence suggests that cytokines in the microenvironment can regulate the polarization state of macrophages, such as IL-4, IL-10, and TGF-β can induce the M2 polarization of macrophages [13, 14]. M2-polarized macrophages accelerated the malignant progression of tumors and promoted the invasion and metastasis of tumors [15]. Cytokines secreted by tumor-associated macrophages (TAMs) promote the development of tumors by forming a complex network, although the specific molecular mechanism is not well understood. M2-type macrophages can induce tumor cell invasion and metastasis by releasing cytokines such as TGF-β and vascular growth factor (VEGF) [14]. In conclusion, we hypothesized that TNFSF9 may affect the occurrence and development of pancreatic cancer by regulating the release of cytokines in the microenvironment.

In the current study, we have studied the effects of TNFSF9 on the proliferation, apoptosis and metastasis of PC cells, as well as the underlying molecular mechanisms. We also studied the effect of TNFSF9 on the polarization state of macrophages in the microenvironment of pancreatic cancer, the effect of polarization state of macrophages on the function of pancreatic cancer cells, and the molecular mechanism of action.

## RESULTS

### TNFSF9 expression is elevated in pancreatic cancer

**Figure 1:**
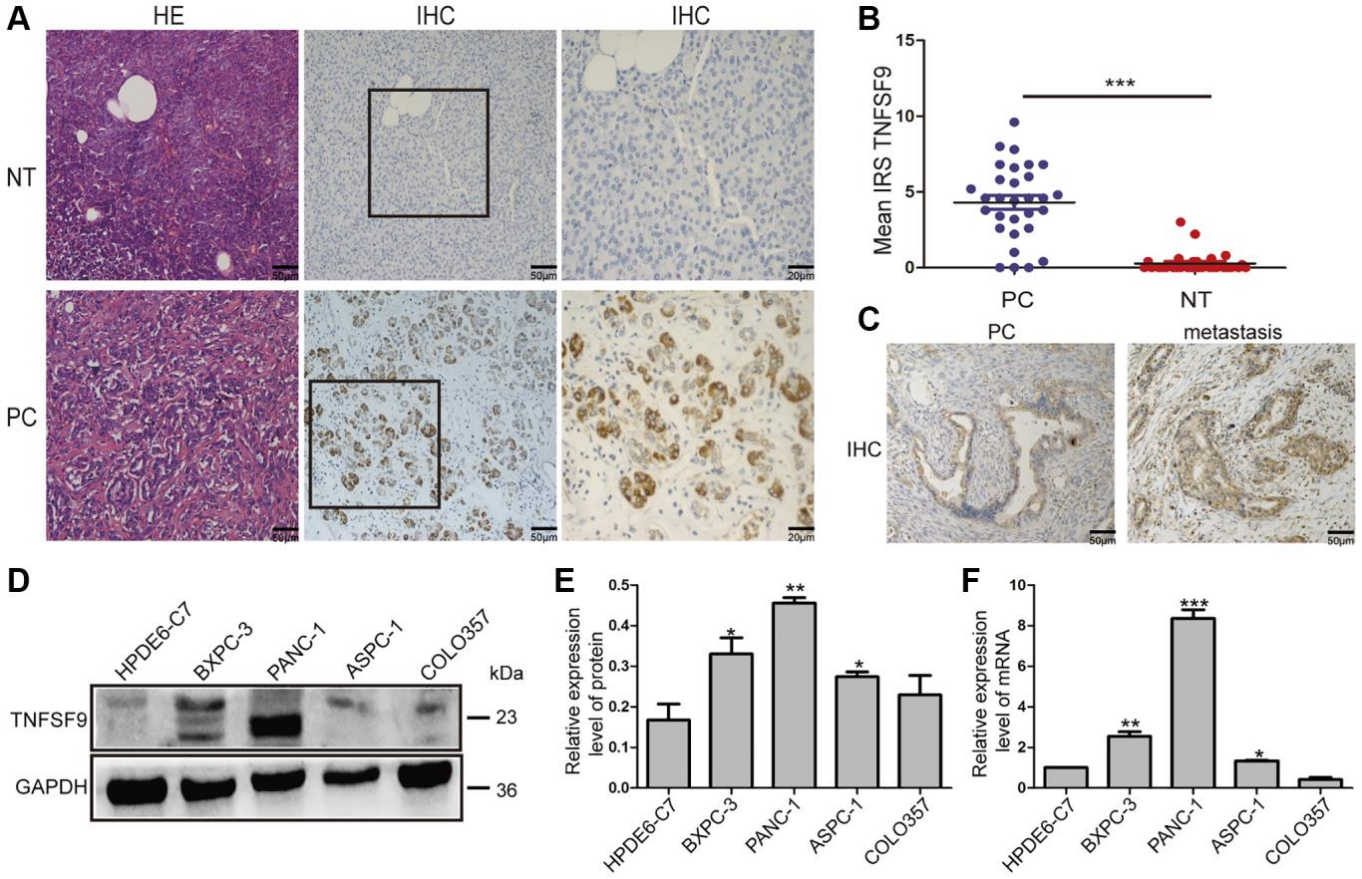
**The expression of TNFSF9 in pancreatic cancer tissues is significantly higher than that in non-tumor tissues adjacent to cancer.** (**A**) Representative HE staining and immunohistochemical staining pictures of cancer tissues and non-tumor tissues adjacent to pancreatic cancer (*n* = 30) and immune response score (**B**). (**C**) Immunohistochemical staining pictures of pancreatic cancer tissue with metastasis and pancreatic cancer tissue without metastasis. (**D**, **E**) Western blot analysis of the protein expression of TNFSF9 in pancreatic cancer cells COLO357, ASPC-1, PANC-1 and BXPC-3 and normal pancreatic epithelial cells HPDE6-C7. (**F**) QPCR analysis of the mRNA expression of TNFSF9 in COLO357, ASPC-1, PANC-1, BXPC-3 and HPDE6-C7. NT, non-tumor tissue adjacent to pancreatic cancer. PC, pancreatic cancer. IRS was used to evaluate tissue staining. The staining intensity is 0 to 3 points, 0 is no staining, 1 is low staining, 2 is medium staining, and 3 is high staining. The percentage of positive cells ranges from 0 to 4 points, 0 < 1%, 1 is 1 to 10%, 2 is 11 to 50%, 3 is 51 to 80%, and 4 is >80%. ^*^P < 0.05, ^**^P < 0.01, and ^***^P < 0.001.

In our previous article, we found that TNFSF9 is positively correlated with the poor prognosis of PC [6]. Therefore, we analyzed the expression of TNFSF9 in PC and normal tissues adjacent to cancer, and found that the expression of TNFSF9 in PC was significantly higher than that in normal tissues adjacent to cancer (*P* < 0.001) (Figure 1A, 1B). Moreover, we found that the expression of TNFSF9 in PC tissues with metastasis was significantly higher than that in PC tissues without metastasis (Figure 1C). In addition, at the mRNA and protein levels, the expression of TNFSF9 in pancreatic cancer cells ASPC-1, PANC-1 and BXPC-3 was significantly higher than that of normal pancreatic epithelial cells HPDE6-C7 (*P* < 0.05), while the expression of COLO357 was not different from HPDE6-C7 (Figure 1D–1F). These results indicated that the expression of TNFSF9 in PC is significantly increased, and may be related to the metastasis of PC.

### TNFSF9 levels correlate with clinicopathological features

In order to study the clinical significance of TNFSF9, we investigated the relationship between TNFSF9 expression and the clinicopathological characteristics of PC patients. As shown in Table 1, the expression of TNFSF9 is associated with lymphatic metastasis (*P* = 0.017) and TNM stage (*P* = 0.003). No differences were found in age, gender, tumor size, or surrounding invasion. These results indicated that TNFSF9 may promote the progress of PC.

**Table 1:** Correlations between TNFSF9 expression and clinicopathological features in pancreatic cancer patients.

**Clinicopathological feature**	**Total**	**Tumoral TNFSF9 expression**		***P* value**
	**(*n* = 30)**	**Low (*n* = 14)**	**High (*n* = 16)**	
Age (years)				
<60	22	12	10	0.226
≥60	8	2	6	
Gender				
Male	15	8	7	0.715
Female	15	6	9	
TNM stage				
Stage I–II	22	14	8	0.003^**^
Stage III–IV	8	0	8	
Tumor size				
<3 cm	22	11	11	0.689
≥3 cm	8	3	5	
Lymph node metastasis				
Positive	9	1	8	0.017^*^
Negative	21	13	8	
Surrounding invasion				
Positive	12	5	7	0.722
Negative	18	9	9	

Note: The bold number represents the *P* values with significant differences. ^*^p < 0.05, ^**^p < 0.01.

### TNFSF9 knockdown inhibits the proliferation of pancreatic cancer cells and promotes the apoptosis of pancreatic cancer cells

**Figure 2:**
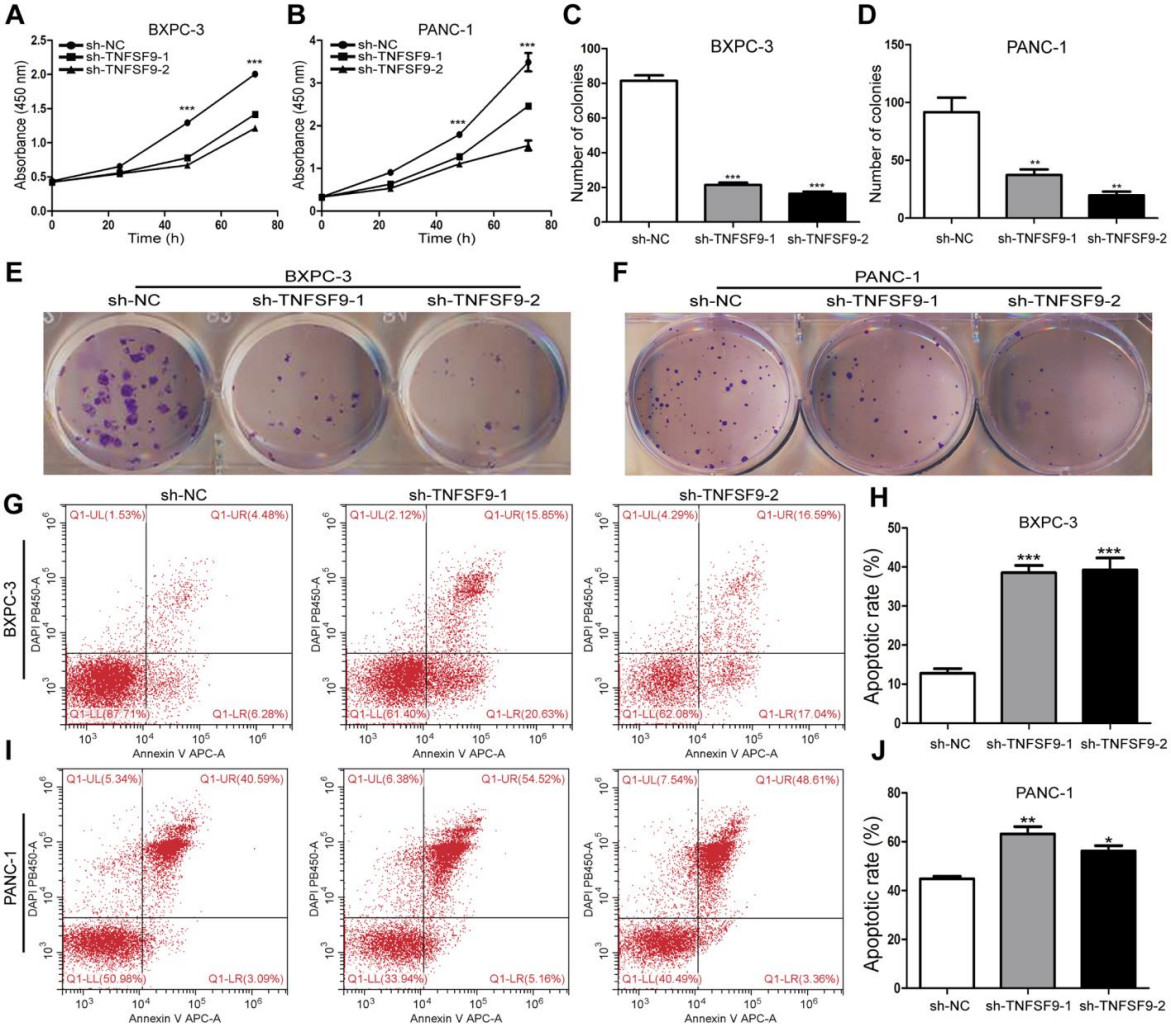
**TNFSF9 knockdown inhibits the proliferation of pancreatic cancer cells and promotes the apoptosis of pancreatic cancer cells.** (**A**, **B**) Cell counting kit-8 was used to evaluate the proliferation of BXPC-3 and PANC-1 cells after TNFSF9 knockdown. (**C**–**F**) Colony formation was used to analyze the proliferation of BXPC-3 and PANC-1 cells after TNFSF9 knockdown. (**G**–**J**) Flow cytometry was used to analyze the apoptosis of BXPC-3 and PANC-1 cells after TNFSF9 knockdown. ^*^P < 0.05, ^**^P < 0.01, and ^***^P < 0.001.

We chose BXPC-3 and PANC-1 cells to construct stable transfected cell lines that knock down TNFSF9. Through the CCK8 experiment, we found that the proliferation of BXPC-3 and PANC-1 cells was inhibited after TNFSF9 knockdown (*P* < 0.05) (Figure 2A, 2B). Similarly, in the single-cell cloning experiment, it was also found that the number of BXPC3 and PANC-1 clones formed after TNFSF9 knockdown was significantly less than that of the sh-NC group (*P* < 0.05) (Figure 2C–2F).

In addition, we also used flow cytometry to analyze the effect of TNFSF9 knockdown on the apoptosis of PC cells, and found that whether it is in BXPC-3 cells or PANC-1 cells, sh-TNFSF9-1 and sh-TNFSF9 -2 compared with the sh-NC group, its apoptosis was significantly increased (*P* < 0.05) (Figure 2G–2J). These results indicated that TNFSF9 can promote the proliferation of PC cells and inhibit the apoptosis of PC cells.

### TNFSF9 knockdown inhibits the migration and invasion of pancreatic cancer cells

**Figure 3:**
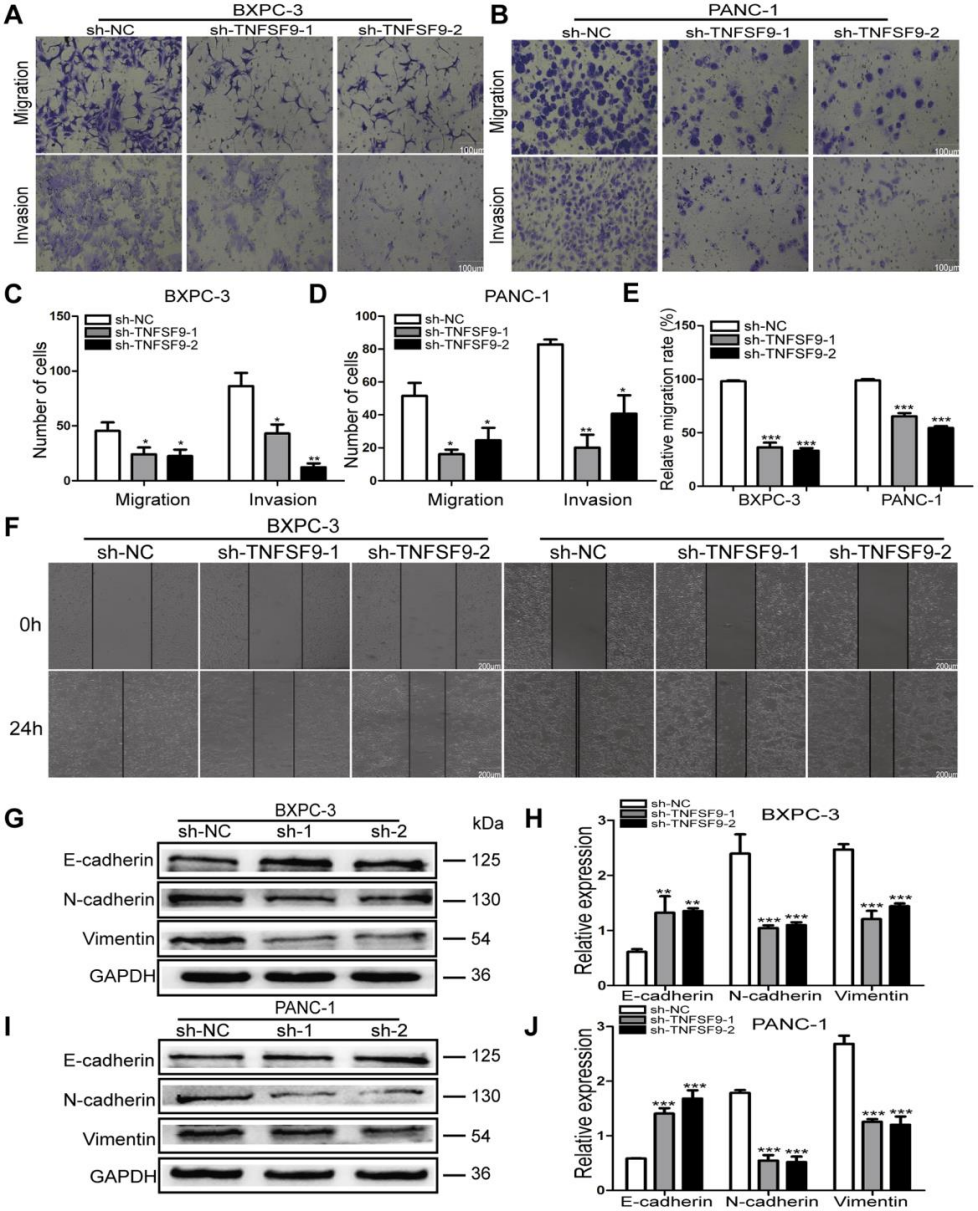
**Knockdown of TNFSF9 inhibits the migration and invasion of pancreatic cancer cells.** (**A–D**) Transwell experiment was used to analyze the migration and invasion of BXPC-3 and PANC-1 cells after TNFSF9 knockdown. (**E**, **F**) The cell scratch test was used to analyze the migration of BXPC-3 and PANC-1 cells after TNFSF9 knockdown. (**G–J**) Western blots were used to analyze the expression levels of EMT-associated proteins; Vimentin, N-cadherin and E-cadherin in BXPC-3 and PANC-1 cells after knockdown of TNFSF9. ^*^P < 0.05, ^**^P < 0.01, and ^***^P < 0.001.

We used transwell experiments to analyze the effect of TNFSF9 knockdown on the migration and invasion of BXPC-3 and PANC-1 cells. We found that after knocking down TNFSF9, the number of migration and invasion of BXPC-3 and PANC-1 cells was significantly reduced compared with the sh-NC group (*P* < 0.05) (Figure 3A–3D). The scratch test also showed that the migration rate of BXPC-3 and PANC-1 cells in the sh-TNFSF9-1 and sh-TNFSF9-2 groups was significantly reduced compared with the sh-NC group (*P* < 0.001) (Figure 3E, 3F). In addition, we analyzed the expression of epithelial-mesenchymal transition (EMT)-related proteins using western blot experiments. We found that the expression of E-cadherin in the sh-TNFSF9-1 and sh-TNFSF9-2 groups was significantly higher than that in the sh-NC group, while the expressions of N-cadherin and Vimentin were significantly lower than those in the sh-NC group (*P* < 0.01) (Figure 3G–3J). It shown that TNFSF9 may promote the metastasis of pancreatic cancer by regulating the EMT process.

### TNFSF9 promotes pancreatic cancer metastasis through Wnt/Snail signaling pathway

**Figure 4:**
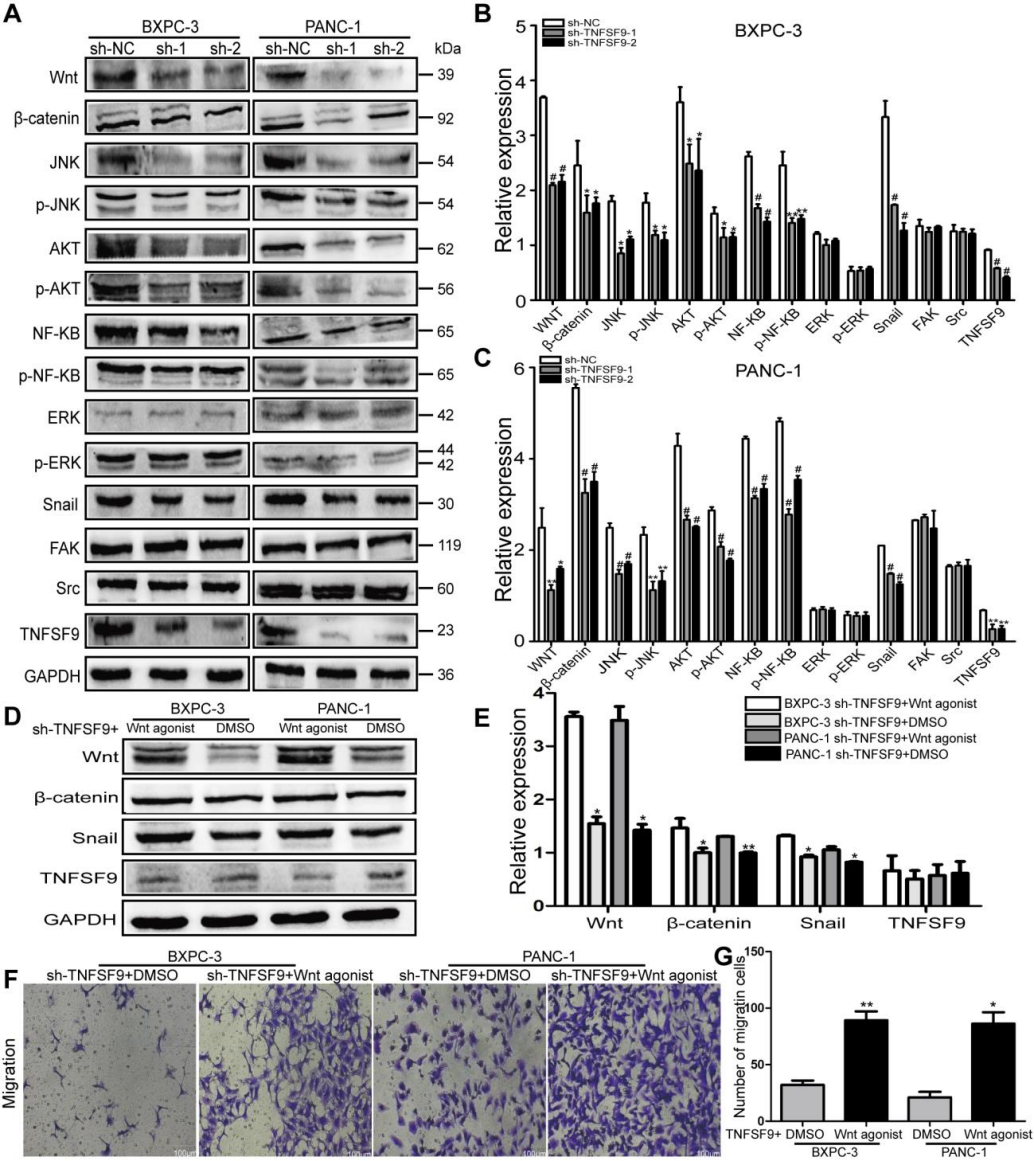
**TNFSF9 promotes the migration of pancreatic cancer cells by activating the Wnt/Snail signaling pathway.** (**A–C**) Western blot analysis the expression of Wnt, β-catenin, AKT, p-AKT, JNK, p-JNK, NF-ΚB, p-NF-ΚB, ERK p-ERK, Snail, Src and FAK in BXPC-3 and PANC-1 cells after knockdown of TNFSF9. Compared with the sh-NC group, the expression of Wnt, β-catenin, AKT, p-AKT, JNK, p-JNK, NF-ΚB, p-NF-ΚB and Snail in the sh-TNFSF9-1 and sh-TNFSF9-2 groups is significantly reduced. The expression of ERK, p-ERK, Src and FAK did not change. (**D**, **E**) The expression of Wnt, β-catenin, and Snail increased after the addition of Wnt agonist in TNFSF9 knockdown pancreatic cancer, but the expression of TNFSF9 did not change. (**F**, **G**) Increased migration of TNFSF9 knockdown pancreatic cancer cells after the addition of Wnt agonist. sh-1 is sh-TNFSF9-1. sh-2 is sh-TNFSF9-2. ^*^P < 0.05, ^**^P < 0.01, ^***^P < 0.001 and ^#^*P* < 0.001.

We analyzed the expression of several signal molecules related to tumor metastasis by western blot. We found that in both BXPC-3 and PANC-1 cells, the expression of Wnt protein in sh-TNFSF9-1 and sh-TNFSF9-2 groups, as well as their downstream β-catenin, Akt, JNK and NF-ΚB was significantly reduced compared with that in sh-NC group. Moreover, the phosphorylation levels of Akt, JNK and NF-ΚB in sh-TNFSF9-1 and sh-TNFSF9-2 groups were also significantly down-regulated compared with the sh-NC group (*P* < 0.05) (Figure 4A–4C). However, there was no significant change in the expression of ERK and p-ERK. In addition, we continued to study the expression of Snail, Src and FAK related to EMT and found that the expression of Snail was significantly down-regulated after TNFSF9 knockdown (*P* < 0.001) (Figure 4A–4C), while the expression of Src and FAK no change.

Furthermore, we added Wnt agonist to TNFSF9 knockdown pancreatic cancer cells, and found that compared with the sh-TNFSF9 + DMSO group, the expressions of Wnt, β-catenin and Snail were increased in the sh-TNFSF9 + Wnt agonist group (*P* < 0.05), while the expression of TNFSF9 was not different (Figure 4D, 4E). In addition, compared with the sh-TNFSF9 + DMSO group, the migration of pancreatic cancer cells in the sh-TNFSF9 + Wnt agonist group was significantly increased (*P* < 0.05) (Figure 4F, 4G). In conclusion, TNFSF9 may promote the metastasis of pancreatic cancer by activating its downstream Wnt/Snail signal.

### TNFSF9 induces M2 polarization of macrophages to promote the metastasis of pancreatic cancer cells

**Figure 5:**
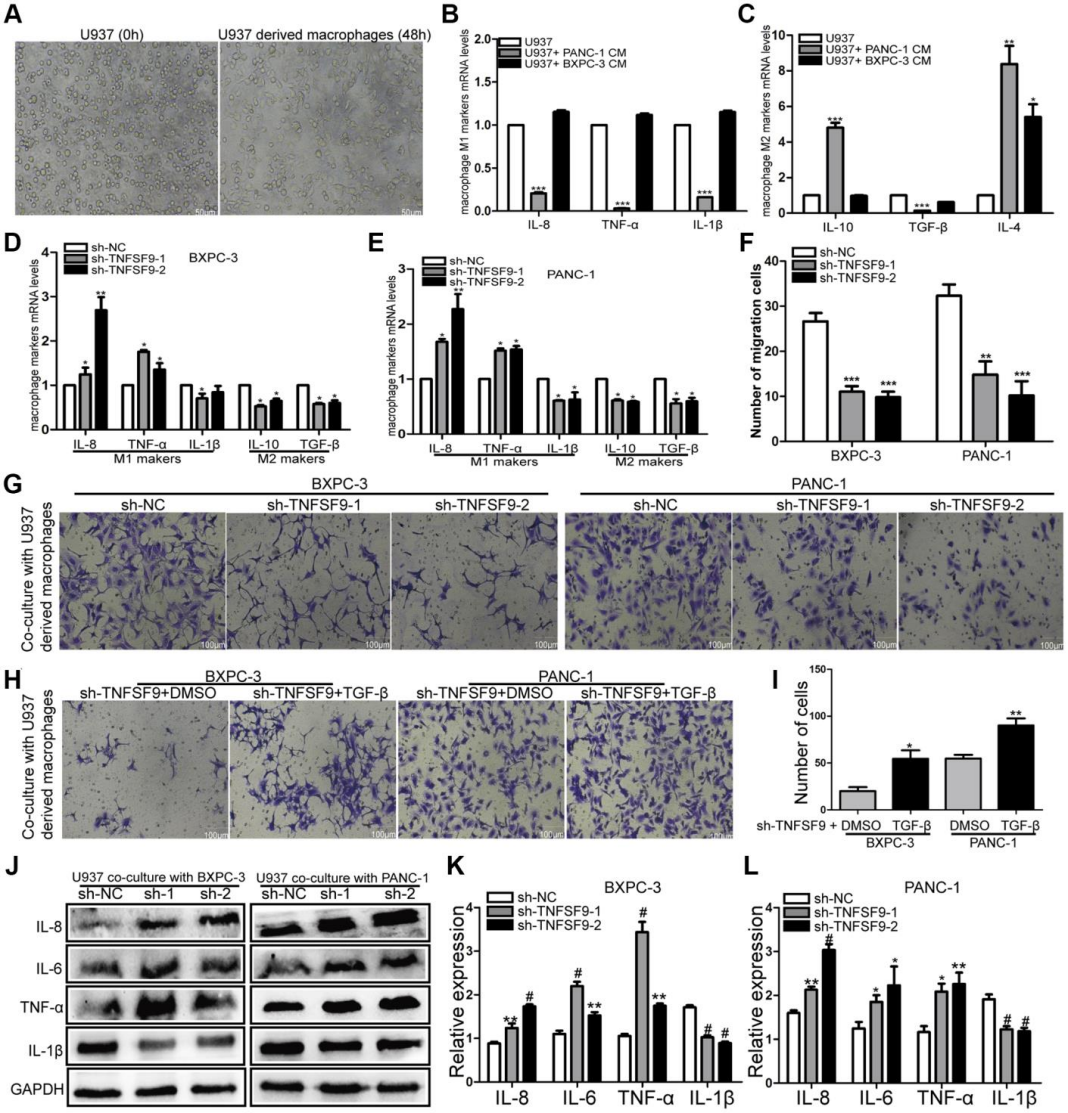
**TNFSF9 induces M2 polarization of macrophages and promotes the migration of pancreatic cancer cells.** (**A**) U937 cells were successfully induced into macrophages. (**B**, **C**) The effect of wild-type pancreatic cancer cells on the polarization of U937-derived macrophages. (**D**, **E**) TNFSF9 knockdown pancreatic cancer cells inhibit the expression of M2 markers (IL-10 and TGF-β) mRNA levels in U937 macrophages. At the same time, it promotes the expression of M1 marker (IL-8 and TNF-α) mRNA level and inhibits the expression of IL-1β mRNA level. (**F**, **G**) Co-culture of U937-derived macrophages with TNFSF9 knockdown pancreatic cancer cells inhibited the migration of BXPC-3 and PANC-1 cells. (**H**, **I**) The migration of BXPC-3 and PANC-1 cells increased after adding recombinant human protein TGF-β. (**J**–**L**) Western blots were used to analyze the expression of inflammatory cytokines IL-8, IL-6, TNF-α and IL-1β on U937-derived macrophages after co-culture with pancreatic cancer cells knocked down by TNFSF9. Compared with the sh-NC group, the expression of IL-8, IL-6 and TNF-α in the sh-TNFSF9-1 and sh-TNFSF9-2 groups were significantly increased, and the expression of IL-1β was significantly reduced. sh-1 is sh-TNFSF9-1. sh-2 is sh-TNFSF9-2. ^*^P < 0.05, ^**^P < 0.01, ^***^P < 0.001 and ^#^*P* < 0.001.

Crosstalk between tumor cells and immune cells can affect tumor metastasis. Therefore, we induced U937 cells into macrophages to analyze the effects of pancreatic cancer cells on macrophages (Figure 5A). We first co-cultured wild-type pancreatic cancer cells with U937-derived macrophages, and found that compared with U937-derived macrophages, mRNA levels of M1-type markers (IL-8, TNF-α and IL-1β) in U937-derived macrophages were significantly decreased after co-cultured with PANC-1 cells (*P* < 0.001) (Figure 5B). The mRNA levels of M2 markers (IL-10, TGF-β and IL-4) were significantly increased (*P* < 0.01) (Figure 5C). BXPC-3 cells only up-regulated IL-4 mRNA levels (*P* < 0.05) (Figure 5C). And then, we co-cultured TNFSF9-knockdown pancreatic cancer cells with U937-derived macrophages and found that compared with the sh-NC group, the mRNA levels of M1-type markers (TNF-α and IL-8) in sh-TNFSF9-1 and sh -TNFSF9-2 groups were increased and the mRNA levels of IL-1β were decreased. MRNA levels of M2-type markers (IL-10 and TGF-β) were decreased (*P* < 0.05) (Figure 5D, 5E). These results suggested that TNFSF9 expression on PC cells can promote M2 polarization of macrophages.

We further co-cultured polarized macrophages with wild-type BXPC-3 and PANC-1, and found that macrophages co-cultured with PC cells knocked down by TNFSF9 inhibited the migration of BXPC-3 and PANC-1 (*P* < 0.001) (Figure 5F, 5G). When the recombinant human protein TGF-β was added, the migration of BXPC-3 and PANC-1 cells increased compared with the TNFSF9 knockdown group (*P* < 0.05) (Figure 5H, 5I). The results showed that M2-polarized macrophages promoted the migration of pancreatic cancer cells by releasing TGF-β.

In addition, we used western blot to verify the expression of inflammatory factors IL-1β, IL-6, IL-8 and TNF-α on co-cultured macrophages. We found that compared with the sh-NC group, the expression of IL-6, IL-8 and TNF-α in the sh-TNFSF9-2 and sh-TNFSF9-2 groups were significantly increased, while the expression of IL-1β was decreased (*P* < 0.05) (Figure 5J–5L). Therefore, we speculated that the high expression of TNFSF9 on PC cells can promote M2 polarization of macrophages, and at the same time, M2 polarized macrophages release related cytokines to promote PC metastasis.

### TNFSF9 activates Wnt signal to promote M2 polarization of macrophages

**Figure 6:**
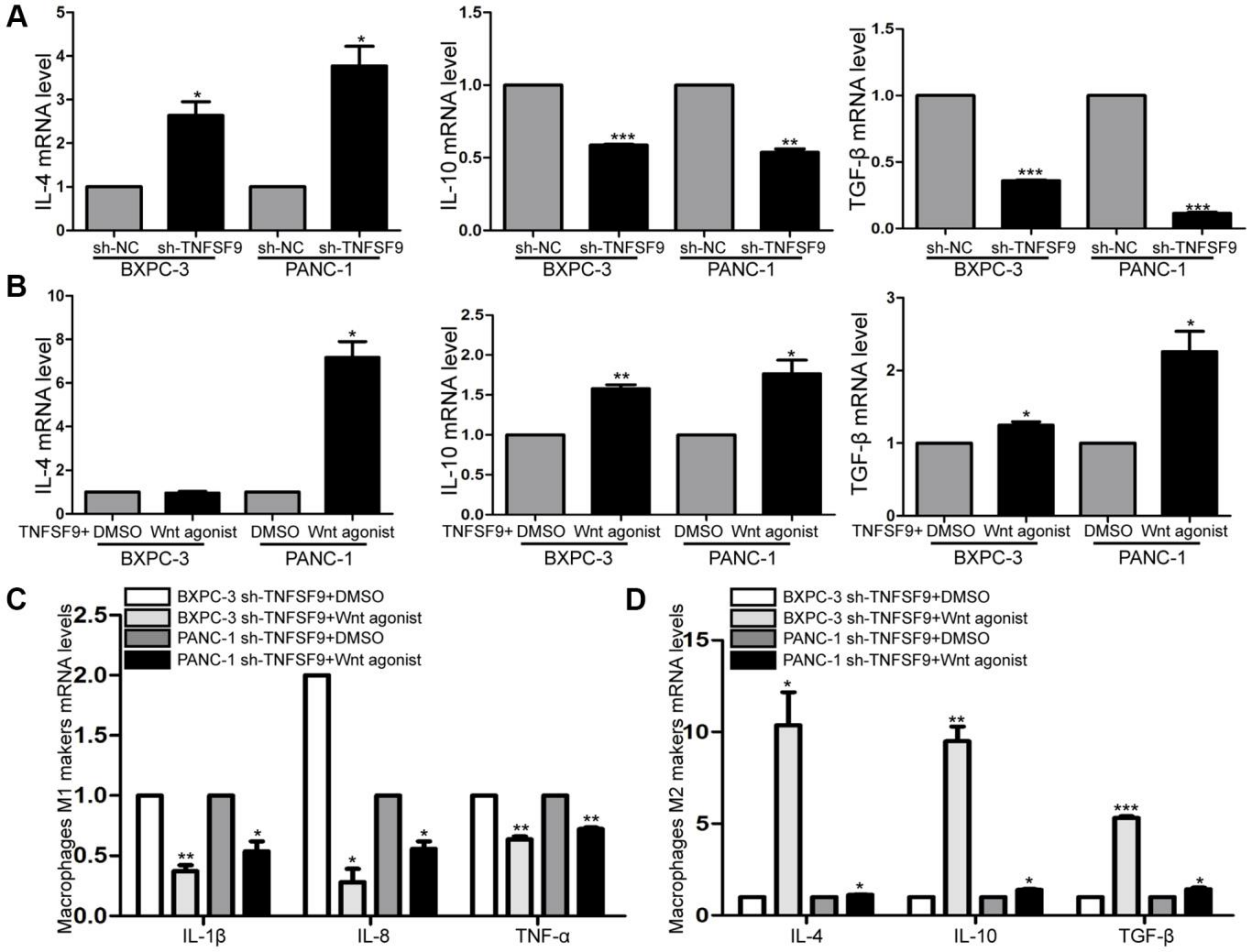
**TNFSF9 activates Wnt signal to promote M2 polarization of macrophages.** (**A**) The mRNA expression levels of IL-10 and TGF-β in TNFSF9 knockdown pancreatic cancer cells were decreased, and the mRNA expression levels of IL-4 were increased. (**B**) After adding Wnt agonist, the mRNA expression level of IL-10 and TGF-β increased. (**C**) After adding Wnt agonist, pancreatic cancer cells that knock down TNFSF9 reduce the mRNA levels of M1 markers (IL-1β, IL-8 and TNF-α) in macrophages. (**D**) After adding Wnt agonist, pancreatic cancer cells that knock down TNFSF9 increase the mRNA levels of M2 markers (IL-4, IL-10 and TGF-β) in macrophages. ^*^P < 0.05, ^**^ *P* < 0.01 and ^***^P < 0.001.

Cytokines secreted by tumor cells can regulate the polarization of macrophages, so we verified the effect of TNFSF9 knockdown on cytokine secretion of tumor cells by qPCR. We found that after TNFSF9 knockdown, the mRNA levels of IL-10 and TGF-β were significantly decreased , while the mRNA levels of IL-4 were increased (*P* < 0.05) (Figure 6A). In addition, we investigated whether Wnt is involved in the regulation of cytokine release, and found that after adding Wnt agonist, the levels of IL-4, IL-10 and TGF-β mRNA in PANC-1 cells that knocked down TNFSF9 were significantly increased. The mRNA levels of IL-10 and TGF-β in BXPC-3 cells were significantly increased (*P* < 0.05) (Figure 6B), while the mRNA level of IL-4 did not change. The results indicated that TNFSF9 may promote pancreatic cancer cells to release IL-10 and TGF-β through Wnt signal to promote M2 polarization of macrophages.

Next, we co-cultured TNFSF9 knockdown pancreatic cancer cells supplemented with Wnt agonist with macrophages, and found that the mRNA levels of M1 macrophage markers (IL-1β, IL-8 and TNF-α) were significantly decreased (*P* < 0.05) (Figure 6C), and M2 markers (IL-4, IL-10 and TGF-β) were significantly increased (*P* < 0.05) (Figure 6D). In summary, TNFSF9 promotes the release of IL-10 and TGF-β from pancreatic cancer cells by activating Wnt signaling, thereby promoting M2 polarization of macrophages.

### TNFSF9 promotes the metastasis of pancreatic cancer *in vivo*

**Figure 7:**
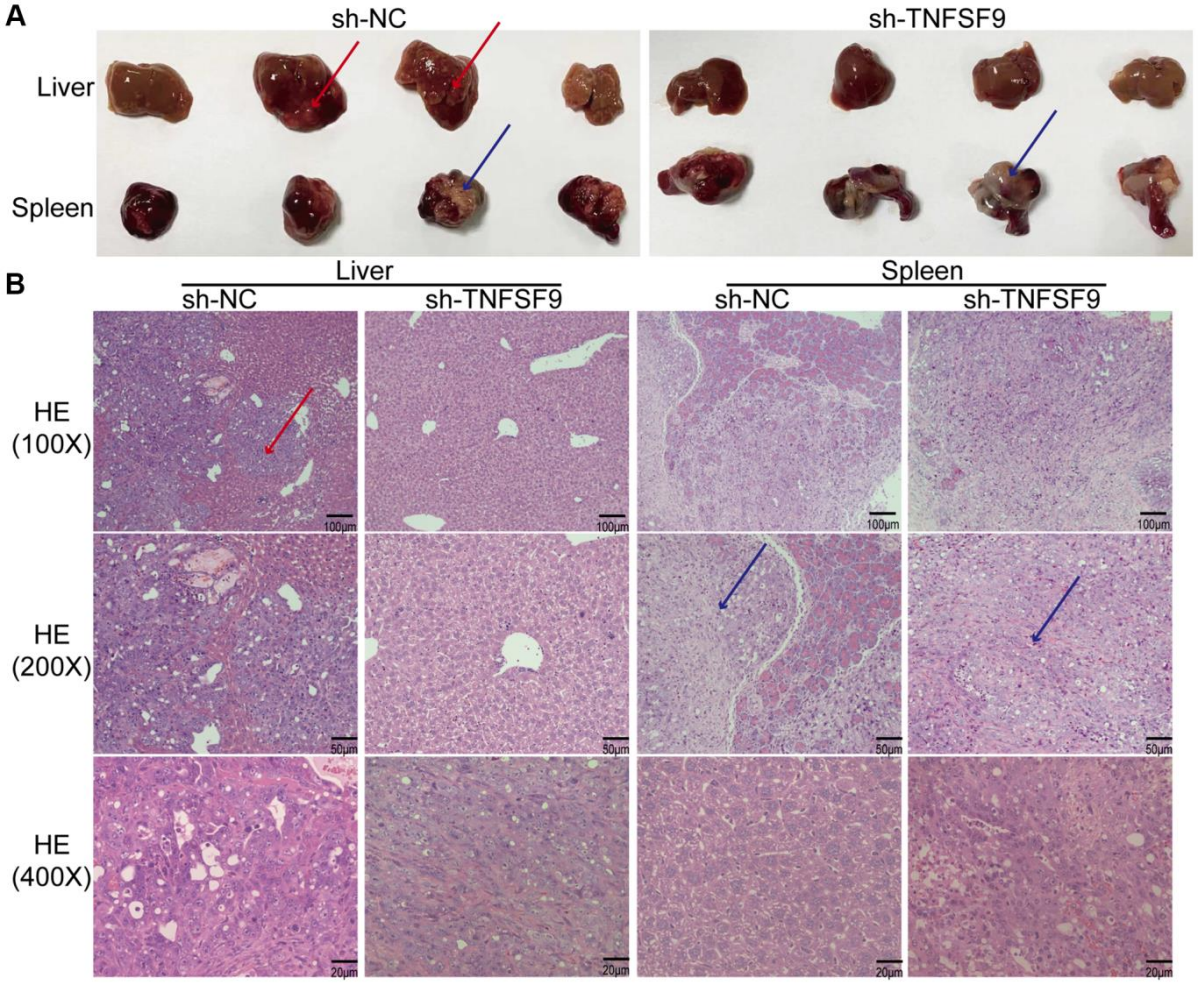
**TNFSF9 promotes metastasis of pancreatic cancer *in vivo*.** (**A**) Compared with the sh-TNFSF9 group, there were obvious metastases in the liver tissue of nude mice in the sh-NC group, and no normal spleen tissue was seen. (**B**) HE staining confirmed liver metastases of pancreatic cancer and splenic implants. The red arrow indicates metastasis of liver lesions. The blue arrow indicates the spleen implanted tumor.

In order to study the effect of TNFSF9 on PC metastasis *in vivo*, we injected TNFSF9 knockdown PANC-1 cells into the spleen of nude mice to construct a metastasis model. We found that all nude mice in the sh-NC group had obvious liver metastasis, with a metastasis rate of 100%, which was manifested as gray-yellow lesions of different sizes on the surface of the liver, which were dispersed and partially fused into blocks. No normal spleen tissue was found, and all were tumor tissues (Figure 7A). Two of the 7 nude mice in the sh-TNFSF9 knockdown group had liver metastases, the metastasis rate was 28.6%, and the metastasis was significantly less than that in the sh-NC group. Normal spleen tissue is still visible (Figure 7A). We sliced liver and spleen tissues and performed HE staining to confirm the results (Figure 7B).

## DISCUSSION

In this article, we found that the expression of TNFSF9 in PC was significantly higher than that of normal tissues adjacent to the cancer, and through *in vitro* cell experiments found that TNFSF9 promotes the proliferation of pancreatic cancer cells and inhibits the apoptosis of pancreatic cancer cells. However, recent data suggest that TNFSF9 reduces the proliferation of hepatocellular carcinoma, small cell lung cancer, and colorectal cancer [8, 10, 16]. This is contrary to the results of our research. Therefore, we hypothesized that TNFSF9 may have individual differences in its effect on tumor cells, showing both inhibitory and activate effects. And, whether TNFSF9 can be used as a specific diagnostic marker for PC due to this difference still needs further study.

The high mortality rate of PC is attributed to the early metastasis [17]. Although TNFSF9 inhibited the proliferation of colorectal cancer cells in colorectal cancer, TNFSF9 also promoted the occurrence of EMT in colorectal cancer cells [8]. In addition, TNFSF9 also promotes bone metastasis of breast cancer by promoting monocyte/macrophage migration and osteoclast differentiation [12]. Therefore, we focused on exploring the effect of TNFSF9 on PC metastasis and demonstrated that TNFSF9 does promote PC metastasis *in vivo* and *in vitro*. And this process may depend on the EMT. EMT is the first step in cancer metastasis, a continuous transition from the pebble epithelial state to the spindle mesenchymal state [18]. EMT confers metastatic properties to cancer cells by enhancing mobility, invasion, and resistance to apoptotic stimuli [19]. Multiple signaling pathways are synergistic in the initiation and development of EMT, such as the TGF-β, Notch, and Wnt signaling pathways [20]. During EMT, Wnt and TGF-β signaling seem to act synergistically to regulate changes in gene expression [21]. TGF-β protein can activate ERK, p38 and Jun MAPK pathways to promote tumor metastasis [22, 23]. Wnt also activates several downstream signaling molecules, such as β-catenin, FAK, ERK, JNK, Src, and Akt, etc. [24]. Therefore, we explored the changes of major signaling pathways after TNFSF9 knockdown, and found that TNFSF9 knockdown down-regulated the expression of Wnt, β-catenin, Akt, JNK, and NF-ΚB, and inhibited the phosphorylation of Akt, JNK, and NF-ΚB. However, there was no significant change in the expression of ERK and p-ERK. Therefore, TNFSF9 may promote metastasis of PC mainly through activation of Wnt and its downstream signaling molecules.

There are many transcription factors involved in the occurrence of tumor EMT, such as Snail/Slug family, Twist, EF1/ZEB1, SIP1/ZEB2 and E12/E47 [25]. Among them, Snail is an important EMT inducer, and the expression of Snail is positively correlated with the tumor grade, tumor recurrence, metastasis and poor prognosis of various malignant tumors [20, 26]. In addition, studies have shown that many signal molecules from the tumor microenvironment can induce the expression of Snail in various cell environments [27], such as extracellular matrix (collagen and hyaluronic acid), soluble factors (epidermal growth factor and fibroblast growth factor), cytokines and other signaling molecules [28]. In addition, Src as a downstream signal of Wnt, has a profound effect on the reorganization of cytoskeleton and adhesion systems that support cell migration and invasion [29]. It mediates many of the processes that tumour cells undergo to gain the ability to invade and spread, while inhibiting Src inhibits the EMT process [30]. Furthermore, the FAK recruits Src and Src substrates to integrin participation sites and plays a role in cell cycle progression and survival as well as adhesion and migration [31]. Therefore, in this study, we explored whether TNFSF9 is involved in metastasis of PC by regulating Snail, Src /FAK. We also confirmed that TNFSF9 may promote PC metastasis through the Wnt /Snail signaling pathway, not dependent on Src/FAK.

In addition, the tumor immune microenvironment promotes multiple aspects of carcinogenesis, including initiation, growth, metastasis, and immune escape. Depending on the tissue of origin, the tumor immune microenvironment displays a unique immune system with different proportions and functional states of lymphocytes, granulocytes, monocytes/macrophages, and dendritic cells (DC) [32]. Among them, macrophages are the most infiltrated immune cells in the tumor microenvironment and are closely related to poor prognosis and treatment failure of tumors [33]. Macrophages are powerful regulators of inflammation and play multiple roles in tumor metastasis [34]. For example, activated macrophages can release inflammatory cytokines, TGF-β, TNF-α, and IL-6 promoted the emergence of EMT [35]. Therefore, after co-culture of TNFSF9 knockdown PC cells and macrophages, we observed the effect of macrophages on the migration of PC cells, and found that the macrophages co-culture with TNFSF9 knockdown PC cells inhibited the migration of PC cells. In addition, previous studies have shown that macrophages can be polarized into a pro-inflammatory M1 phenotype and an anti-inflammatory M2 phenotype according to the tumor microenvironment [36]. Among them, M2 type macrophages are closely related to tumor metastasis. M2 macrophage derived exosomes promote metastasis of colon cancer [37]. Tumor-recruited M2 macrophages promote metastasis of gastric cancer and breast cancer by secreting CHI3L1 protein [38]. After M2 polarization, the release of anti-inflammatory cytokines IL-10 and IL-4 was increased, while the release of pro-inflammatory cytokines IL-6, IL-1β and TNF-α was decreased, and these cytokines regulate the occurrence of EMT [35, 39]. In this article, we further found that the M2 polarization of macrophages co-cultured with TNFSF9 knockdown pancreatic cancer cells was reduced, and this result was dependent on the activation of Wnt signaling by TNFSF9 to promote the release of IL-10 and TGF -β in pancreatic cancer cells. After the M2 polarization of macrophages decreases, the expression of pro-inflammatory cytokines IL-6, IL-8 and TNF-α increases, while the expression of IL-1β decreases. Therefore, we hypothesized that the high expression of TNFSF9 in PC cells could not only directly promote the metastasis of pancreatic cancer, but also promote the metastasis of PC by inducing M2 polarization of macrophages and releasing some cytokines.

In conclusion, our results indicate that the expression of TNFSF9 is significantly elevated in PC, which may promote the growth and metastasis of PC *in vivo* and *in vitro* through the Wnt /Snail signaling pathway. In addition, TNFSF9 can induce the M2 polarization of macrophages by activating the Wnt signal of pancreatic cancer cells to secrete cytokines, thereby promoting the metastasis of pancreatic cancer. Therefore, our study not only identified a new biomarker for pancreatic cancer, but also provided a new target for the combination therapy of pancreatic cancer.

## MATERIALS AND METHODS

### Patient sample collection

From August 2017 to December 2019, we collected 30 cases of paraffin-embedded pancreatic cancer and normal adjacent tissues from the Department of Pathology of Chongqing Medical University. Written informed consent was obtained from all patients according to institutional guidelines. The study was approved by the Ethical Review Committee of Chongqing Medical University.

### Cell culture and transfection

Human pancreatic cancer cell lines ASPC-1 and COLO357 were purchased from the Shanghai Chinese Academy of Sciences Cell Bank (Shanghai, China). Other cell lines, PANC-1, BXPC-3 and HPDE6-C7 were donated by Dr. Tan Peng from Southwest Medical University. PANC-1, BXPC-3, COLO357 and HPDE6-C7 were cultured in high glucose DMEM containing 10% fetal bovine serum (Pan Biotech, Germany). ASPC-1 was cultured in 1640 medium containing 10% fetal bovine serum. All cells were cultured under 5% CO2, 37°C and humidified conditions [40].

Lentiviruses targeting TNFSF9 (sh-TNFSF9-1, 5′-CGCCACAGTCTTGGGACTCTT-3′ and sh-TNFSF9-2, 5′-TGGAATACGCCTCTGACGCTT-3′) and negative control viruses (sh-NC, 5′-TTCTCCGAACGTGTCACGT-3′) were purchased from GeneChem (Shanghai, China). BXPC-3 and PANC-1 cells were transfected according to the manufacturer's instructions, and the knockdown efficiency was evaluated by western blot.

### Preparation of conditioned medium and cell co-cultivation

Stably transfected BXPC-3 and PANC-1 cells were seeded into a six-well plate at a density of 1 × 10^5^, cultured for 24 hours, and then replaced with a serum-free medium for 48 hours. The supernatant was collected and centrifuged at 3000 rpm for 5 minutes to remove cell debris to obtain conditioned medium (CM) [41].

The human monocyte cell line U937 was cultured in 1640 medium containing 10% fetal bovine serum, and 100 ng/mL phorbol 12-myristate 13-acetate (PMA) (Sigma-Aldrich, St. Louis, Missouri, USA) was added at 37°C. After 48 hours, a macrophage-like phenotype was obtained. CM was added to the U937-derived macrophage cell line and cultured for 24 hours. The cells were collected for subsequent experiments [41].

### RNA extraction and quantitative real-time PCR analysis

Perform the qPCR experiment as described above [36]. In brief, total RNA was obtained by Trizol reagent (Takara, Japan) according to the manufacturer's instructions. Then PrimeScript RT kit (Takara, RR037A) was used for the reversal transcription. Finally, TB Green Premix Ex TaqII (Takara, RR820A) was used for qPCR analysis. The relative expression levels were calculated using 2^−Δ∆ct^ method. The primers used are as follows: GAPDH, forward, 5′-CACCACCCTGTTGCTGT-3′, and reverse, 5′-CCACTCCTCCACCTTTG-3′; IL-8, forward, 5′-AAGAAACCACCGGAAGGAAC-3′, and reverse, 5′-ACTCCTTGGCAAAACTGCAC-3′; TNF-α, forward, 5′-AGATGGGAAGGGAATGAACC-3′, and reverse, 5′-GACGTGTCACGATCATCTGG-3′; IL-10, forward, 5′-CGAGATGCCTTCAGCAGAGTG-3′, and reverse, 5′-TCATCTCAGAACAAGGCTTGGC-3′; transforming growth factor-β (TGF-β), forward, 5′-CAACACATCAGAGCTCCGAGAA-3′, and reverse, 5′-GCTGAGGTATCGCCAGGAAT-3′, IL-1β forward, 5′-GAAATGATGGCTTATTACAGTGGC-3′, and reverse, 5′-TTGCTGTAGTGGTGGTCGGAG-3′; IL-4, forward, 5′-CAGTTCCACAGGCAAAGCA-3′, and reverse, 5′-CATGATCGTCTTTAGCCTTTCC-3′.

### Immunohistochemistry

Immunohistochemistry was performed on paraffin-embedded tissues. The specific experimental method is as described above [42]. TNFSF9 (1:100, ProteinTech) was used to incubate tissues at 4°C overnight. After the application of appropriate secondary antibodies, the labeled antigens were visualized using the standard 3,3′-diaminobenzidine (DAB) protocol. The slide is then back-dyed with hematoxylin. Immunoreactivity score (IRS) was used to evaluate tissue staining. The staining intensity is 0 to 3 points, 0 is no staining, 1 is low staining, 2 is medium staining, and 3 is high staining. The percentage of positive cells ranges from 0 to 4 points, 0 < 1%, 1 is 1 to 10%, 2 is 11 to 50%, 3 is 51 to 80%, and 4 is >80%. Multiplying the average intensity and percentage of positively stained cells produces an IRS, with a maximum score of 12 points. These data represent the average of the results obtained by the two scorers.

### Cell proliferation analysis

We use Cell Counting Kit-8 (CCK8) to detect cell proliferation [43]. Stably transfected PANC-1 and BXPC-3 cells were seeded into 96-well plates at a density of 2000 cells per well, and cultured under 5% CO2 and 37°C. The CCK8 reagent was added at 0, 24, 48, and 76 hours, and the absorbance at 450 nm was detected with a microplate reader (Bio-Rad Laboratories, Inc.).

The single-cell cloning experiment is as described above. The stably transfected PANC-1 and BXPC-3 cells were seeded into a 6-well plate at a density of 500 cells per well, and cultured under the conditions of 5% CO2 and 37°C for 2 weeks. Cells were fixed with 4% paraformaldehyde for 20 minutes and stained with 1% crystal violet for 25 minutes. The number of clones (>50 cells/colony) was calculated using an inverted microscope (Leica, Germany).

### Apoptosis analysis

For apoptosis analysis, the stably transfected PANC-1 and BXPC-3 cells were first digested with trypsin, washed twice with PBS, and resuspended in 100 ul PBS. Cells were then stained in the dark with AnnexinV-fluorescein isothiocyanate (FITC)/PI kit (BD Biosciences, USA) for 15 min, and apoptosis was detected by flow cytometry (BD Biosciences, San Jose, CA, USA) [43].

### Cell migration and invasion analysis

Cell migration and invasion were analyzed using a 0.8 um chamber (Corning Inc., Corning, NY, USA) [43]. For cell migration analysis, 5 × 10^5^ stably transfected PANC-1 and BXPC-3 cells were resuspend in 200 ul serum-free medium and seeded into the upper chamber, and 500 ul DMEM medium containing 10% FBS was added into the lower chamber and cultured at 5% CO_2_ at 37°C for 48 h. Cells were fixed with 4% paraformaldehyde for 20 min and stained with 1% crystal violet for 25 min. Five random fields were selected for cell count using an inverted microscope (Leica Microsystems, Germany). For cell invasion analysis, Matrigel (BD Bioscience) was first diluted with DMEM in a ratio of 1:8. 100 ul of the mixture was added to the upper chamber and incubated at 37°C for 2 h to solidify. The other experimental steps were the same as the transfer analysis.

For cell scratch analysis, stably transfected BXPC-3 and PANC-1 cells were segregated into 6-well plates. When the degree of cell confluence reached 90%, scratches of the same width were made with the tip of a 10 ul sterile pipette, and images were obtained immediately with a microscope. After incubation in serum-free medium for 24 h, the scratches were recorded again.

### Western blot analysis

First, the total cell protein was extracted with protein extract (Beyotime, China). Then, 10% sodium dodecyl sulfate polyacrylamide gel (SDS-PAGE) was used for electrophoresis, and the protein was transferred to polyvinylidene (PVDF) and sealed with 5% skimmed milk for 1 h. It was then incubated overnight at 4°C with the following primary antibodies: GAPDH (1:10000, ProteinTech), TNFSF9 (1:2000, ProteinTech), β-catenin (1:1000, ProteinTech), Wnt (1:1000, Wanleibio), ERK (1:500, Wanleibio), phosphorylated-ERK (1:1000, Santa), JNK (1:1000, Wanleibio), phosphorylated-JNK (1:1000, Wanleibio), AKT (1:1000, Santa), phosphorylated-AKT (1:1000, Santa), NF-ΚB (1:1000, Wanleibio), phosphorylated-NF-ΚB (1:1000, Wanleibio), Snail (1:1500, Wanleibio), focal adhesion kinase (FAK) (1:2000, ProteinTech), proto-oncogene tyrosine-protein kinase Src (Src) (1:800, ProteinTech), IL-6 (1:5000, Wanleibio), IL-8 (1:1000, Wanleibio), TNF-α (1:1000, Santa), E-cadherin (ProteinTech, 1:10000), N-cadherin (ProteinTech, 1:3000), and Vimentin (ProteinTech, 1:2000). Finally, the membrane was incubated with appropriate secondary antibodies for 1 h at room temperature. The band was quantitatively measured using the Zeiss LSM800 spectrometer (Carl Zeiss AG, Germany) [44].

### Establishment of metastasis model in nude mice

At 5 weeks of age, female BALB/c nude mice (7 in each group) were purchased from the National Laboratory Animal Center (Shanghai, China) and reared under standard pathogen-free conditions. Stably transfected PANC-1 cells were resuspended in 100 ul PBS, and the cell concentration was adjusted to 5 × 10^6^/mL. After intraperitoneal injection of 1% sodium pentobarbital (50 mg/kg), the nude mice were placed in the right decubitus position and disinfected with 75% alcohol. A longitudinal incision was made in the left flank of the abdomen with a length of about 1.0 cm. The spleen was exposed and gently pulled out of the abdominal cavity. The lower pole of the spleen was inserted with a needle of about 1.0 cm, and the tumor cell suspension was slowly injected. After the injection, the needle was quickly pulled out and pressed on the eye of the needle for 2 minutes with an iodophor cotton ball. Full belly closure. Routine feeding of mice after surgery. After cultured for 4 weeks, the mice were sacrificed by cervical dislocation and dissected to observe the tumor formation of spleen, liver metastasis and metastasis in other parts. The spleen transplanted tumor and liver metastatic tumor were collected and stained with hematoxylin-eosin (HE) [45].

### Data statistics

Statistical analysis was performed with GraphPad Prism 8.0 and SPSS23.0 software. All data are presented as mean ± standard deviation (SD) for at least three independent trials. The data were analyzed using one-way ANOVA, and then multiple comparisons were performed using Bonferroni's test. ^*^P < 0.05 was considered statistically significant.
